# Metabolic Syndrome Risk after Gestational Diabetes: A Systematic Review and Meta-Analysis

**DOI:** 10.1371/journal.pone.0087863

**Published:** 2014-01-31

**Authors:** Yuhong Xu, Shutong Shen, Lizhou Sun, Haiwei Yang, Bai Jin, Xiaohui Cao

**Affiliations:** 1 Department of Gynecology and Obstetrics, the First Affiliated Hospital of Nanjing Medical University, Nanjing, People's Republic of China; 2 Nanjing Medical University, Nanjing, People's Republic of China; 3 Clinical Research Center, the First Affiliated Hospital of Nanjing Medical University, Nanjing, People's Republic of China; Azienda Policlinico S. Orsola-Malpighi, Italy

## Abstract

**Background:**

A number of studies have been conducted to investigate the risk of metabolic syndrome (MS) after gestational diabetes mellitus (GDM), but the results are contradictory. Accordingly, we performed a systematic review and meta-analysis to assess the association between these two conditions. The aim was to better understand the risks of MS with prior gestational diabetes.

**Methods:**

Pubmed, ISI Web of Science, and Cochrane databases from September 1, 1979 to July 11, 2013 were searched to identify relevant studies. 17 studies containing 5832 women and 1149 MS events were included. We calculated the odds ratio (OR) with 95% confidence interval (CI) in analysis for each study using a random-effect or fixed-effect model. We also determined heterogeneity among these 17 articles and their publication bias.

**Results:**

Women with a history of gestational diabetes had a significantly higher risk of MS than those who had a normal pregnancy (OR, 3.96; 95% CI, 2.99 to 5.26), but had significant heterogeneity (*I* ^2^ = 52.6%). The effect remained robust (OR, 4.54; 95% CI, 3.78–5.46) in the subgroup of Caucasians, but no association (OR, 1.28; 95% CI, 0.64–2.56) was found in Asians. Heterogeneity was reduced (body mass index (BMI) matched group *I* ^2^ = 14.2%, BMI higher in the GDM group *I* ^2^ = 13.2%) in the subgroup of BMI. In addition, mothers with higher BMI in the GDM group had higher risk of MS than those in the BMI matched group (BMI higher in GDM group OR, 5.39; 95% CI, 4.47–6.50, BMI matched group OR, 2.53; 95% CI, 1.88–3.41).

**Conclusions:**

This meta-analysis demonstrated increased risk of MS after gestational diabetes. Therefore, attention should be given to preventing or delaying the onset of MS in GDM mothers, particularly in Caucasian and obese mothers.

## Introduction

Gestational diabetes mellitus (GDM) is defined as carbohydrate intolerance that begins or is first recognized during pregnancy [1]. It complicates about 1% to 14% of all pregnancies worldwide [2], [3], resulting in large health care and economic costs. The disease develops mainly in the second half of gestation as a result of insulin resistance that is thought to be induced by excessive placenta hormones. Normal glucose tolerance is usually restored after delivery. However, GDM mothers and their offspring are at increased risk of developing type 2 diabetes mellitus (T2DM) in the future [4], [5]. They are also at an increased risk of cardiovascular disorders (CVDs) such as hypertension and coronary artery diseases [6]. A screening program at 6 weeks postpartum has been recommended for affected women, but most do not attend the program [7]. Years later, they become part of the diabetic or CVD population, posing a heavier burden to society.

Metabolic syndrome (MS) is considered as the concomitant clustering of central obesity, hypertriglyceridemia, low high-density lipoprotein (HDL) cholesterol, hypertension, and dysglycemia [8], [9]. In recent decades, the prevalence of MS has rapidly increased as a result of unhealthy lifestyles. Although the etiology and diagnostic criteria of MS is still a subject of debate, the condition shares common features with GDM, including insulin resistance, dyslipidemia, and endothelial dysfunction [10]. Moreover, the clinical impact of MS has been considered a cause of cardiovascular morbidity and mortality [11]. Based on these similarities, a growing number of studies have investigated the relationship between GDM and MS. Several studies observed an increased risk of MS in association with a history of GDM [10], [12], while others not [13], [14]. Accordingly, we performed a comprehensive systematic review and meta-analysis in this work to assess the overall risk of women with prior GDM developing MS. We also conducted subgroup analysis to investigate the effect of factors that may modify this risk.

## Materials and Methods

### Literature search strategy

A preliminary search was conducted in Pubmed, ISI Web of Science, and Cochrane database from September 1, 1979 to July 11, 2013 without population and language restriction. The search term combinations were “gestational diabetes”, “metabolic syndrome”, “insulin resistance syndrome” and “syndrome X”. All reference lists from the main reports and relevant reviews were hand searched for additional eligible studies.

### Eligible studies and data extraction

Eligible studies had to meet the following criteria: (1) retrospective or prospective studies, (2) original papers with independent data, (3) content of diagnostic criteria of GDM and MS, and (4) outcome studied was the risk of MS at least 6 weeks after pregnancy. We excluded studies if any of the following applied: (1) case-only or cross-sectional studies, (2) overlapping data, and (3) review articles and letters. If different diagnostic criteria of MS were found in a study, our first choice was the National Cholesterol Education Program Adult Treatment Panel (NCEP-ATP III), and our second choice considered International Diabetes Federation (IDF). If data from different follow-up years were contained in a study, we chose data for the longest follow-up year.

Data extraction was independently performed by two reviewers. Eligible studies from the two reviewers were compared for any inconsistency, and disagreement was settled by further discussion among all authors. For each included study, a specific data extraction form was created to collect the following information: first author's surname, study type, publication year, country, number of MS from GDM, number of MS from control, GDM criteria, MS criteria, and mean follow-up year.

### Statistical methods

The risk of MS after GDM was analyzed by calculating the odds ratio (OR) with 95% confidence interval (CI). Heterogeneity occurred when more variations between studies than expected by chance were found. Thus, *I* ^2^ was measured to assess heterogeneity as a measure of percentage of variability in effect estimate because of heterogeneity rather than sampling error. In the absence of significant heterogeneity (*I* ^2^<50%), a fixed-effect model was used. A random-effect model was used if substantial heterogeneity was detected (*I* ^2^>50%). We investigated possible sources of identified heterogeneity by subgroup analysis according to study design (prospective or retrospective studies), ethnic origin (Asian, Caucasian, or mixed), maternal age (matched, higher in GDM group, or higher in control group), body mass index (BMI) during follow-up (matched or higher in GDM group), GDM criteria (World Health Organization (WHO), Carpenter and Coustan, Canadian Diabetes Association Guidelines, Danish, National Diabetes Data Group (NDDG), or American Diabetes Association (ADA)), MS criteria (NCEP-ATP III or IDF), number of incident cases (<50, 50–100, or >100), and mean follow-up year (<1 year, 1 to 5 years, or >5 years). Publication bias was assessed using Begger's funnel plot. All analyses were carried out using Stata software version 11.0. Statistical significance was set at *P*<0.05, and 95% CIs were quoted throughout.

## Results

**Figure 1:**
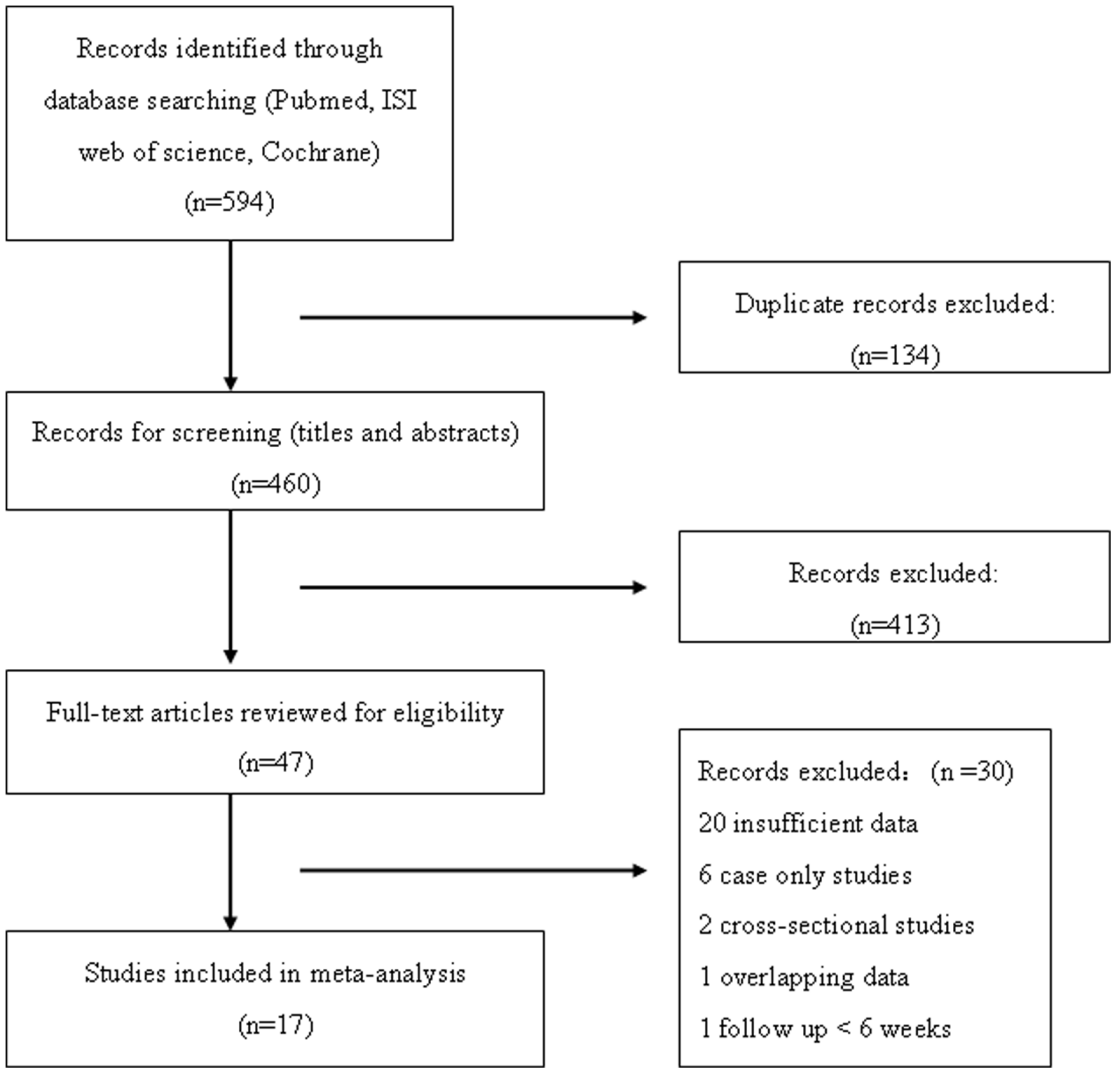
Flow diagram of study selection.

Figure 1 shows the details of the selection process (reasons for exclusion are listed). A total of 594 records were identified through electronic database searches. After excluding duplicates, 460 were screened (titles and abstracts) and 47 were selected for full-text reading. Another 30 studies were excluded because of various reasons as listed in Figure 1, and 17 studies were included in the meta-analysis. Table 1 presents the main characteristics of the 17 included studies containing 2520 cases and 3312 controls [10], [12]–[27]. Fourteen of the studies (82.35%) were prospective in design, whereas the other 3(17.65%) were retrospective. Thirteen studies (76.47%) were of Caucasian origin, and only two (11.76%) were of Asian origin. Diversity was noteworthy as far as GDM diagnostic criteria were concerned. Criteria from WHO and Carpenter and Coustan were more frequently used in studies, whereas CDA Guidelines, Danish, NDDG, and ADA criteria were each applied in only one study.

**Table 1:** Characteristics of the studies included in this meta-analysis.

study source	study type	year	country	MS/GDM	MS/control	GDM criteria	MS criteria	mean follow up years
Edalat [17]	retrospective study	2013	Iran	23/77	9/67	WHO	NCEP-ATP III	2–3 years
Tam [13]	prospective study	2012	China	10/45	14/94	WHO 1999	IDF	15 years
Akinci [12]	prospective study	2011	Turkey	49/195	4/71	Carpenter and Coustan	NCEP-ATP III	GDM 3.38 years/control 3.39 years
Retnakaran [15]	prospective study	2010	Canada	27/137	26/259	Canadian Diabetes Association Guidelines	IDF	3 months
Madarasz [16]	prospective study	2009	Hungary	18/68	3/35	WHO 1985	NCEP-ATP III	4 years
Costacou [18]	prospective study	2008	USA	10/22	8/29	ADA	NCEP-ATP III	GDM 2.1 years/control 2.3 years
Wender-Ozegowska [19]	prospective study	2007	Poland	47/153	8/155	WHO	NCEP-ATP III	GDM 6.0 years/control 5.1 years
Tam [14]	prospective study	2007	China	5/67	11/136	WHO 1999	IDF	8 years
Krishnaveni [20]	prospective study	2007	India	21/35	125/489	Carpenter and Coustan	IDF	5 years
Di Cianni et [10]	prospective study	2007	Italy	15/166	1/98	Carpenter and Coustan	NCEP-ATP III	16 months
Wijeyaratne [21]	prospective study	2006	Sri Lanka	72/147	4/67	WHO	IDF	3 years
Bo [22]	retrospective study	2006	Italy	34/182	4/161	Carpenter and Coustan	NCEP-ATP III	6.5 years
Kousta [23]	retrospective study	2006	England	136/368	48/482	WHO	IDF	20 months
Lauenborg [24]	prospective study	2005	Danish	199/457	146/987	Danish	NCEP-ATP III	9.8 years
Albareda [25]	prospective study	2005	Spain	29/262	4/66	NDDG	NCEP-ATP III	5 years
Bo [26]	prospective study	2004	Italy	17/81	3/65	Carpenter and Coustan	NCEP-ATP III	8.5 years
Verma [27]	Prospective study	2002	USA	15/58	4/51	Carpenter and Coustan	NCEP-ATP III	11 years

WHO: World Health Organization; ADA: America Diabetes Association; NDDG: National Diabetes Data Group; NCEP-ATP III: National Cholesterol Education Program Adult Treatment Panel; IDF: International Diabetes Federation.

**Figure 2:**
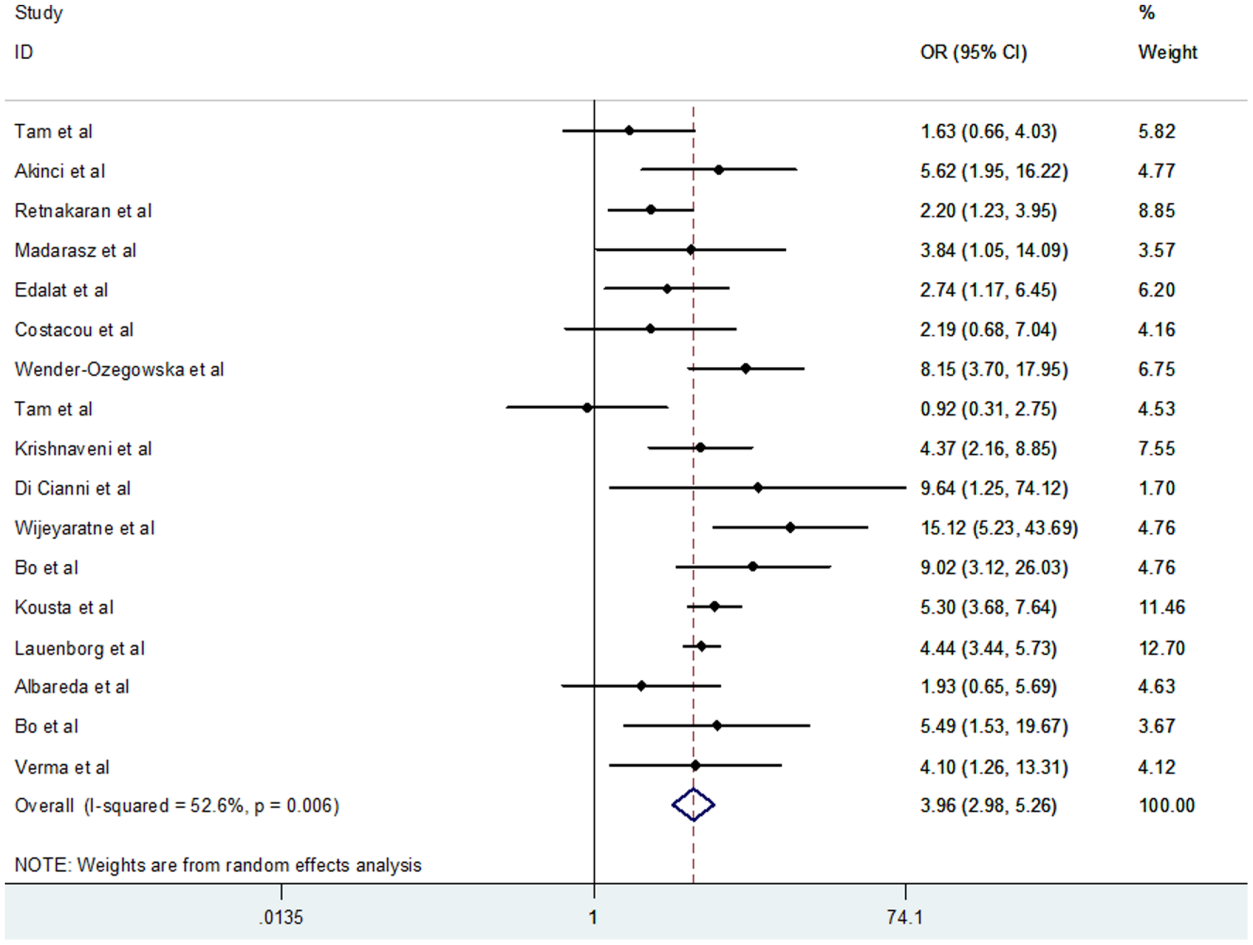
Forest plot of overall risk of metabolic syndrome (MS) after gestational diabetes mellitus (GDM).

**Figure 3:**
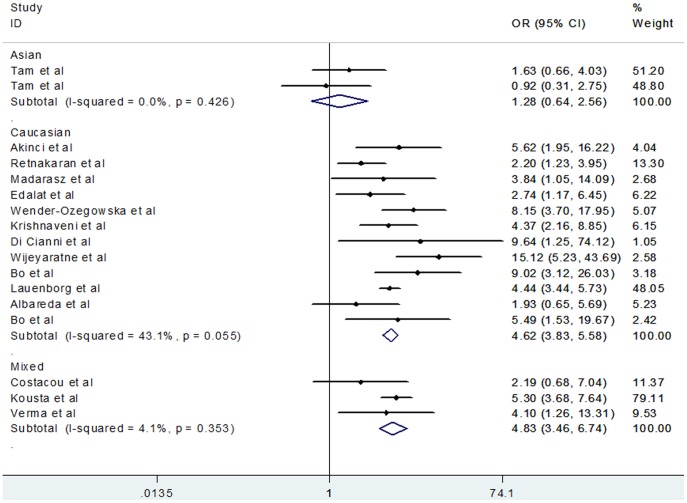
Forest plot of risk of metabolic syndrome after gestational diabetes mellitus grouped by Ethnic origin.

**Figure 4:**
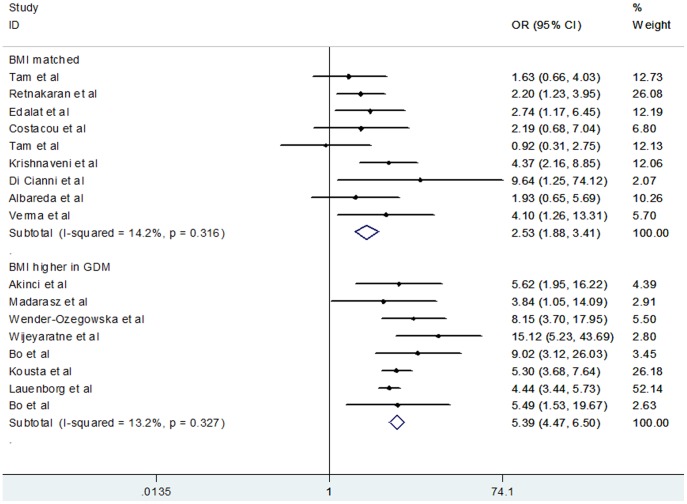
Forest plot of risk of metabolic syndrome after gestational diabetes mellitus grouped by body mass index (BMI).

Figure 2 shows that women with previous GDM demonstrated significantly higher risk of developing MS, with evidence of heterogeneity in the risk estimate (random effects OR, 3.96; 95% CI, 2.98–5.26, *P*<0.001; heterogeneity *I* ^2^ = 52.6%). A priori hypothesis to explain potential heterogeneity among studies included study type, ethnic origin, maternal age, BMI, GDM criteria, MS criteria, number of incident cases, and mean follow-up year (Table 2). Study type, maternal age, GDM criteria, MS criteria, number of incident cases, and mean follow-up year failed to provide indications of effect on the chances of developing MS in women with a history of GDM. However, the outcome significantly differed when ethnicity was used as a subgroup factor. Figure 3 shows that Caucasian women demonstrated a significantly higher chance of developing MS after diabetic pregnancy than Asian women (Caucasian OR, 4.54; 95% CI, 3.78 to 5.46; Asian OR, 1.28; 95% CI, 0.64 to 2.56). Moreover, in the subgroup of BMI studies, heterogeneity was reduced and the result was distinctive between subgroups (Figure 4). Specifically, women with prior GDM had higher chances of MS when they owned a larger BMI (BMI higher in GDM group OR, 5.39; 95% CI, 4.47 to 6.50; heterogeneity *I* ^2^ = 13.2%; BMI matched group OR, 2.53; 95% CI, 1.88 to 3.41; heterogeneity *I* ^2^ = 14.2%).

**Table:** **Table 2.** Subgroup analysis of the risk of MS after GDM.

	Studies	MS/GDM	MS/control	OR	95% CI	*I* ^2^ (%)
**Study type**						
prospective study	14	534/1893	361/2602	3.71	2.63∼5.26	54.9
retrospective study	3	193/627	61/710	5.18	3.76∼7.13	37.2
**Ethnic origin**						
Asian	2	15/112	25/230	1.28	0.64∼2.56	0
Caucasian	13	561/1982	345/2549	4.54	3.78∼5.46	41.9
mixed	2	151/426	52/533	5.17	3.65∼7.34	0
**Maternal age**						
matched	13	442/1807	140/1646	3.60	2.39∼5.41	60.50
higher in GDM	3	86/256	136/679	5.65	3.47∼9.19	0
higher in control	1	199/457	146/987	4.44	3.44∼5.73	—
**BMI**						
matched	9	155/869	202/1289	2.53	1.88∼3.41	14.20
higher in GDM	8	572/1651	220/2023	5.39	4.47∼6.50	13.20
**GDM criteria**						
WHO	7	311/925	97/1036	3.86	2.11∼7.06	73.10
Carpenter and Coustan	6	151/717	141/935	5.75	3.72∼8.89	0
Canadian Diabetes Association Guidelines	1	27/137	26/259	2.2	1.23∼3.95	—
Danish	1	199/457	146/987	4.44	3.44∼5.73	—
NDDG	1	29/262	4/66	1.93	0.65∼5.69	—
ADA	1	10/22	8/29	2.19	0.68–7.04	—
**MS criteria**						
NCEP-ATP III	11	456/1721	194/1785	4.59	3.73∼5.63	0
IDF	6	271/799	228/1527	4.07	3.19∼5.19	78.4
**Number of incident cases**						
<50	10	176/1028	61/802	3.15	2.25∼4.40	36.0
50∼100	4	195/632	42/552	5.91	2.40∼14.52	77.90
>100	3	356/860	319/1958	4.70	3.85∼5.75	0
**Mean follow up year**						
<1 year	1	27/137	26/259	2.20	1.23∼3.95	—
1–5 years	9	373/1340	206/1404	4.89	3.78∼6.33	31.40
>5 years	7	327/1043	190/1649	3.88	2.30∼6.55	63.9

**Figure 5:**
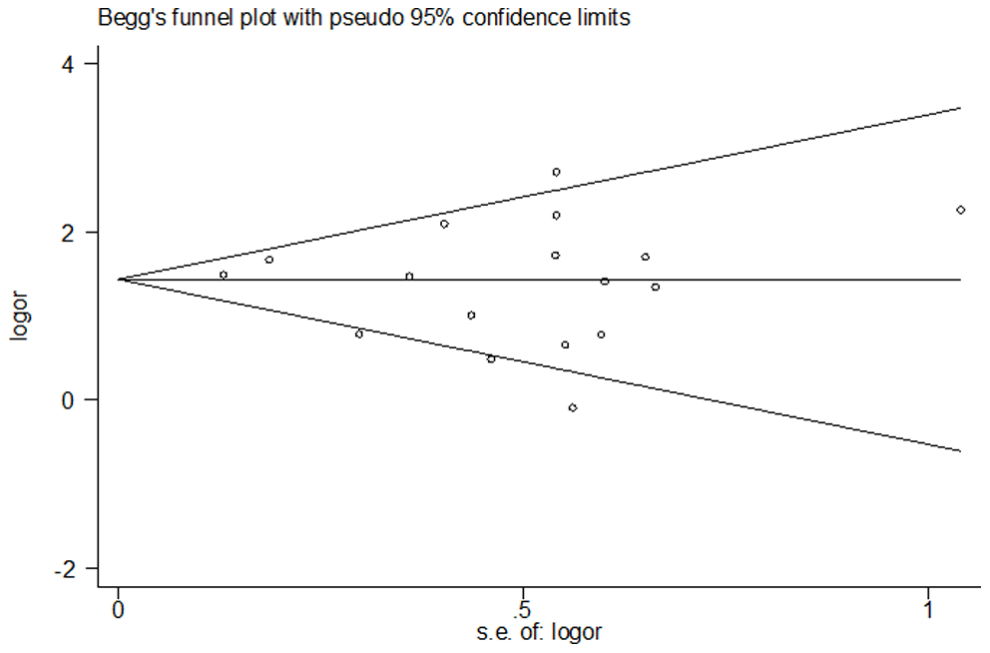
Begger's test indicating no obvious publication bias of 17 studies included in this meta-analysis.

Publication bias was identified by Begger's funnel plot (Figure 5). The shape of this funnel plot was symmetrical, indicating the absence of obvious publication bias (*P* = 0.61).

## Discussion

The incidence of T2DM and MS is rising rapidly worldwide because of the prevalence of unhealthy diet and physical inactivity [28], [29], [30]. In 2005, Ford [31] reported that MS is associated with twofold to threefold increased risk of T2DM and premature CVDs. A number of studies have confirmed this observation [32], [33]. These reports highlighted the importance of MS screening and prevention. As a metabolic disorder, GDM is considered to be similar to DM wherein pregnancy acts as a “stressor.” A meta-analysis conducted in 2009 demonstrated that women who have had GDM have at least sevenfold increased risk of developing T2DM in the future than those who have had normal pregnancy [34]. To the best of our knowledge, GDM shares several common risk factors (family history of DM, increased age, and raised BMI) with T2DM and similarly with MS. Women with MS in early pregnancy have a greater risk of developing GDM [35], whereas GDM mothers are more susceptible to MS during pregnancy [36]. These results indicate that the two diseases may have similar pathogenesis, especially genetic background. A relationship among the risk gene variants of TCF7L2 (rs7903146), FTO (rs9939609), and GDM has also been found in MS [37], [38], [39], thereby supporting our hypothesis. Apart from these studies, researchers from different countries have devoted themselves to investigating the risk of MS after GDM, with conflicting results. In our meta-analysis, we demonstrated that women with a history of GDM had nearly fourfold increased risk of developing MS in the future than those who had normal pregnancy. We supposed that the diagnosis of GDM may act as a precursor or a signal of various metabolic diseases in the near future. Therefore, the correct time (during pregnancy and postpartum) should be determined for GDM women to take steps to decrease the risk of MS, thereby decreasing the risk of T2DM and CVDs. To the best of our knowledge, the development of MS after gestational glucose disorders has not been given as much attention as the increased risk of T2DM. The increased risk of MS reported in this meta-analysis may also provide clinicians and GDM mothers with knowledge on the risks of GDM and ensure that screening programs can be successfully carried out after pregnancy.

Inflammation is considered to be the missing link between GDM and MS, which is associated with insulin resistance [40]. A high level of c-reactive protein (CRP) is believed to induce chronic inflammation, as well as the levels of uric acid, a marker for the risk of CVD and T2DM. Di Cianni et al. [10] reported that after diabetic pregnancy, insulin resistance, serum uric acid, and CRP were significantly higher in women with MS compared with those without MS. Osteoprotegerin (OPG), a novel soluble member of tumor necrosis factor receptor superfamily, has been linked with CVDs. Women with previous GDM who have developed MS exhibited higher osteoprotegerin levels than those without MS [41]. Furthermore, Akinci et al. [42] showed that pre-pregnancy obesity, weight gain during follow-up, and fasting glucose level during the oral glucose tolerance test (OGTT) of the index pregnancy are predictors of developing MS. Generally, fasting glucose levels greater than 100 mg/dL at OGTT of the index pregnancy is an independent predictor of MS development. According to these studies, we suggested that serum fasting glucose, CRP, uric acid, and OPG, together with weight and height measures, should be included in the postpartum screening program for GDM women as a forecast of MS.

Heterogeneity was noted in the overall effect estimate. Accordingly, we conducted subgroup analysis to seek the potential source. Ethnicity may significantly affect MS susceptibility. Here, we did not find any association between GDM and MS in Asians, which can be due to the smaller number of cases of Asians and thus requires further analysis. The ethnicity factor may due to genetic variance and environment effect. When BMI was used as a subgroup factor, heterogeneity was reduced. Therefore, we considered BMI as a confounder in the overall risk estimate. Another result was that GDM women with higher BMI were more susceptible to MS after childbirth, indicating that obese women had more chance of developing MS than non-obese women after diabetic pregnancy. This may because adipose tissues plays an important role in the regulation of insulin sensitivity through secreting adipocytokines, which are involved in the pathogenesis of pregnancy-induced insulin resistance [43].

Several limitations of this meta-analysis should be discussed. Considering the limited information in relevant studies, we were unable to perform subgroup analyses according to family history of diabetes, previous diabetic pregnancy, smoking, pregnant frequency, diabetes prior to GDM and the treatment procedures of GDM. Moreover, as a result of non-informed GDM diagnostic criteria, we included studies with all GDM criteria, which may have some influence on overall risk evaluation. As we previously supposed, the risk of MS after GDM increased as years passed, but the results were not very satisfactory. Although the risk of MS after one to five follow-up years was larger than that after 1 year, limited studies on the risk within 1 year follow-up should be considered. The risk of MS after 5 years decreased when compared with that after 1–5 years, and heterogeneity was much larger. We attributed this result to Asian studies. Both studies were above five years and from the same author, which may have some influence on the result. We were aware of the limitations of observational studies as the main source of evidence as well as the inherent bias associated with experimental design. However, studies on randomized controlled trials concerning the risk of MS after GDM are limited.

The advantages of this meta-analysis should likewise be considered. First, the study elucidated the association of MS after GDM, providing a reason for clinicians to encourage GDM mothers to participate in a screening program after delivery. This practice is definitely beneficial to our society and GDM women themselves. Second, substantial numbers of cases and controls were pooled from multiple databases, which significantly increased the statistical power of the analysis. Third, we found no publication bias in this meta-analysis; thus, this study was credible.

In summary, this meta-analysis provided evidence for an increased risk of MS after GDM, supporting the hypothesis that GDM may be a susceptible marker of MS. Considering obesity as a risk factor of MS, GDM women should pay more attention to controlling their weight. Lifestyle modifications including dietary habits and exercise continue to be the cornerstone [44]. Large sample studies are warranted to validate our findings especially in Asian populations. More relevant randomized controlled trials and intervention studies should also be considered in future to better understand the risks of MS after GDM.

## Supporting Information

Checklist S1 **PRISMA checklist.**
(DOC)
